# Mitochondrial dysfunction and oxidative stress mediate the physiological impairment induced by the disruption of autophagy

**DOI:** 10.18632/aging.100038

**Published:** 2009-04-09

**Authors:** J. Julie Wu, Celia Quijano, Edmund Chen, Hongjun Liu, Liu Cao, Maria M. Fergusson, Ilsa I. Rovira, Sarah Gutkind, Mathew P. Daniels, Masaaki Komatsu, Toren Finkel

**Affiliations:** ^1^ Translational Medicine Branch, National Heart Lung and Blood Institute, NIH, Bethesda, MD 20892, USA; ^2^ NHLBI Electron Microscopy Core Facility, National Heart Lung and Blood Institute, NIH, Bethesda, MD 20892, USA; ^3^Laboratory of Frontier Science, Tokyo Metropolitan Institute of Medical Sciences, Tokyo, Japan

**Keywords:** aging, mitochondria, oxidative stress, autophagy, Atg7

## Abstract

Impaired or deficient autophagy is believed to cause or contribute to aging, as well as a number of age-related pathologies. The exact mechanism through which alterations in autophagy induce these various pathologies is not well understood.  Here we describe the creation of two *in vivo* mouse models that allow for the characterization of the alteration in mitochondrial function and the contribution of the corresponding oxidative stress following deletion of Atg7. Using these models we demonstrate that isolated mitochondria obtained from Atg7^-/-^ skeletal muscle exhibit a significant defect in mitochondrial respiration.  We further show that cells derived from Atg7^-/-^ mice have an altered metabolic profile characterized by decreased resting mitochondrial oxygen consumption and a compensatory increase in basal glycolytic rates. Atg7^-/-^cells also exhibit evidence for increased steady state levels of reactive oxygen species. The observed mitochondrial dysfunction and oxidative stress is also evident in a mouse model where Atg7 is deleted within the pancreatic β cell. In this model, the simple administration of an antioxidant can significantly ameliorate the physiological impairment in glucose-stimulated insulin secretion.  Taken together, these results demonstrate the potential role of mitochondrial dysfunction and oxidative stress in autophagy related pathology.

## Introduction

There is a growing interest in the role of macroautophagy, herein simply termed autophagy, in both normal homeostasis and in a variety of pathological conditions [1,2]. This interest has been sparked in part by observations suggesting that in lower organisms, autophagy is an important determinant of lifespan [3]. For instance, in *C elegans*, the life extending effects of mutation in the *daf-2* pathway requires an intact autophagy program [4,5]. Similarly, in the worm, the increase in lifespan seen with dietary restriction is not evident when autophagy is impaired [6]. Consistent with these observations, genetic manipulations that can increase autophagy in *Drosophila* result in flies with an extended lifespan and an increase in overall stress resistance [7].

Less is known regarding the role of autophagy in mammalian systems. Prior to the establishment of the molecular and biochemical basis for autophagy, it was well appreciated that aging tissues were often characterized by the accumulation of damaged cellular components. In addition, consistent with a defect in autophagy, it was also evident that animal tissues exhibited an age-dependent decline in the turnover rates of long lived proteins [8]. These and other studies have suggested that autophagic flux declines with age and that the magnitude and timing of this decline is in general concordance with the age-dependent accumulation of damaged proteins and organelles seen within aging tissues. Recent genetic mouse models have strengthened this association. While complete knockouts of essential autophagy genes appear to be lethal in the neonatal stage, various conditional knockout models have been recently described. Among these recent results are the description of tissue specific deletions of essential autophagy genes in liver, brain, pancreas and heart [9-14]. While significant differences exist in these various model systems, most were characterized by the rapid appearance of various pathologies and physiological impairments that can also be observed as a consequence of normal aging.

Relatively little is known about the downstream mediators of the often profound physiological alterations observed following the disruption of autophagic flux. Most likely there is no single pathway across all tissues and organs, and even within a single tissue type, multiple mediators may exist. For instance, while the accumulation of misfolded and aggregated proteins normally cleared in part by autophagy are likely to play a prominent role in models of neurodegeneration, in other tissues, the role of accumulation of damaged protein aggregates is less clear. This tissue specificity was reinforced by recent observations demonstrating that deletion of p62, a ubiquitin and LC3/Atg8 binding protein, rescues the pathological changes observed in autophagy deficient liver but does not appear to alter the phenotypic changes seen following deletion of Atg7 in the brain [15].

One important function of autophagy is the turnover of organelles including mitochondria. While several reports have described the structural changes in mitochondrial appearance evident in electron micrographs taken of autophagy deficient mammalian tissues [9,12-14], the functional alterations, if any, of these mitochondria have not been reported. Here, using a variety of cellular and *in vivo *models of Atg7 deficiency, we have assessed the magnitude of mitochondrial dysfunction and the contribution of the corresponding oxidative stress in mediating the physiological impairment observed following disruption of autophagic flux.

## Results

### Mitochondrial dysfunction in Atg7 deficient skeletal muscle

**Figure 1.**
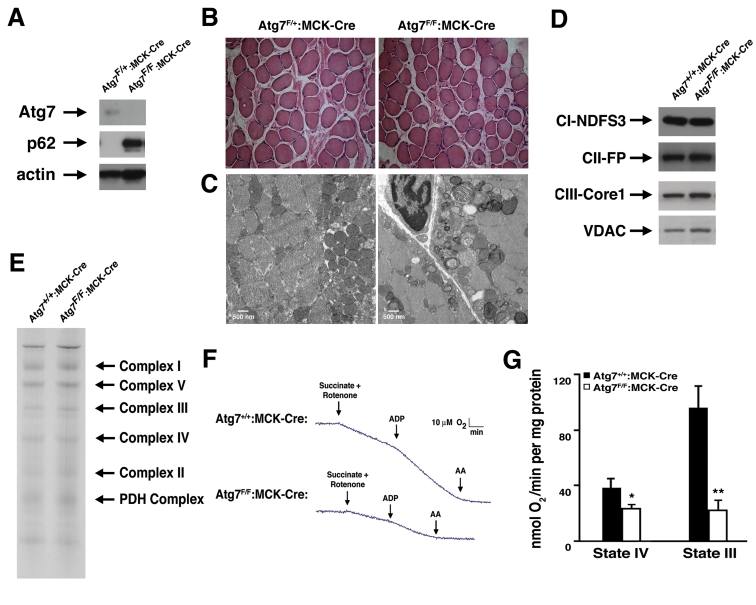
Impaired mitochondrial function in Atg7 deficient skeletal muscle. (**A)** Western blot analysis of protein lysate obtained from mice with a conditional deletion of Atg7 within skeletal muscle (Atg7^F/F^:MCK-Cre) or control animals (Atg7^F/+^:MCK-Cre). Relative levels of Atg7, p62 and actin (loading control) were assessed. (**B)** Representative stained histological sections from soleus muscle.  (**C)** Electron micrographs of skeletal muscle from 8 week old Atg7^F/+^:MCK-Cre or Atg7^F/F^:MCK-Cre mice.   Electron micrographs demonstrate the accumulation of dysmorphic mitochondria within the Atg7^F/F^:MCK-Cre muscle. (**D**) Western blot analysis of purified mitochondria obtained from Atg7^F/F^:MCK-Cre or control animals Atg7^+/+^:MCK-Cre demonstrating an apparent similar level of electron transfer subunit composition. Mitochondrial protein lysates were probed for the Complex I component protein NDFS3 (NADH-ubiquinone oxidoreductase 30 kDa subunit), the Complex II component FP (flavoprotein subunit of complex II ), the Complex III protein Core 1 (Ubiquinol-cytochrome-c reductase complex core protein 1)  and the mitochondrial membrane protein VDAC.  (**E**) Blue Native gel electrophoresis using mitochondrial extracts isolated from control and Atg7^-/- ^tissues demonstrating similar stoichiometry and assembly of electron transfer complex components. (**F**) Representative oxygen consumption tracings of mitochondria isolated from the skeletal muscle of 12 month old Atg7^+/+^:MCK-Cre or Atg7^F/F^:MCK-Cre mice. Atg7-deficient skeletal muscle demonstrated a pronounced reduction in respiration when assessed in the presence of the Complex II dependent substrate succinate. Rotenone was routinely added to prevent reverse electron transport. Measurements were made in the absence (State IV) and presence (State III) of ADP and following the Complex III inhibitor antimycin A (AA). (**G**) Composite determinations of mitochondrial respiration in the presence of succinate and rotenone. Graph represents the mean +/- SEM from Atg7^+/+^:MCK-Cre (n=3) or Atg7^F/F^:MCK-Cre mice (n=3). * p≤0.05; ** p≤0.01.

In an effort to more fully characterize the role of autophagy in the maintenance of normal mitochondrial function, we created a conditional knockout model in which Atg7, an essential gene required for autophagosome formation, was deleted from mouse skeletal muscle. We initially chose this model because skeletal muscle is an abundant tissue that is also a rich source of mitochondria. As expected, muscle protein lysate from animals expressing the Cre recombinase under the control of the muscle creatine kinase (MCK) promoter demonstrated reduced Atg7 expression when compared to skeletal muscle tissue obtained from control mice (Figure 1A). Coincident with a reduction in Atg7 expression, we noted a marked increase in the level of p62, a protein cleared in large part through autophagy and whose levels are routinely used as a marker of overall autophagic flux [16]. Although these results suggest that autophagy in skeletal muscle was largely impaired in these conditionally ablated animals, when compared to control animals, we observed no obvious differences in terms of viability, overall size and activity, serum parameters or generalized appearance of the Atg7^F/F^:MCK-Cre mice throughout the first year of life (unpublished observations). In addition, histological analysis revealed no discernable skeletal muscle structural abnormalities between knockout and control mice (Figure 1B). In contrast, electron micrographs demonstrated that Atg7^F/F^:MCK-Cre mice accumulated markedly abnormal mitochondria that were especially evident in the sub-sarcolemma region of muscle fibers. These abnormal mitochondria appeared less electron dense and were often swollen, lacking in cristae, or dysmorphic in appearance (Figure 1C). Despite these profound differences in mitochondrial appearance, we observed no obvious alterations in the composition of the various cytochrome complexes (Figure 1D), nor were there obvious differences in the assembly of individual electron transfer components using Blue Native Gel analysis (Figure 1E).

In order to test whether the observed alterations in mitochondrial appearance were also accompanied by corresponding functional changes, we measured the basal and stimulated respiration of mitochondria isolated from Atg7^F/F^:MCK-Cre and control animals. Preliminary experiments suggested that while Complex I dependent respiration was reduced, Complex II dependent respiration was even more impaired in Atg7 deficient muscle.  Using succinate as a substrate, mitochondrial function was noted to be markedly reduced when we tested mitochondria isolated from Atg7 deficient animals (Figure 1F). These defects were evident under basal conditions (State IV) and even more so, under conditions of maximal stimulated respiration (State III) induced by the addition of ADP (Figure 1G).

### Atg7^-/-^ MEFs demonstrate impaired cellular respiration and increased ROS levels

**Figure 2.**
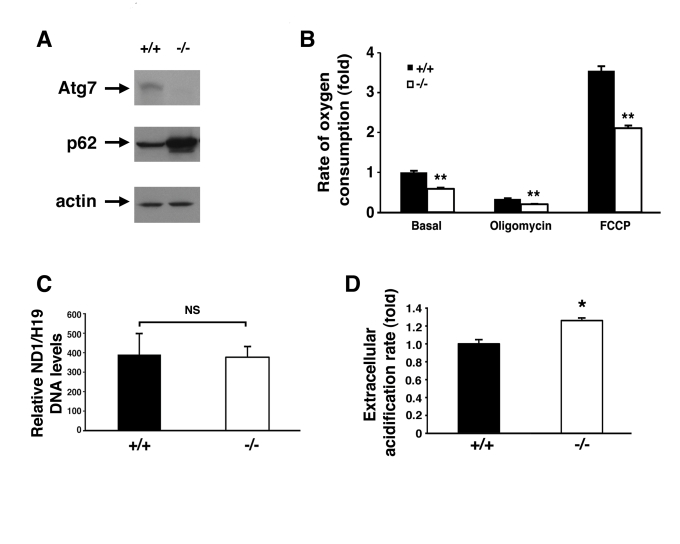
Alterations in the energetics of Atg7 ^-/-^ MEFs. (**A**) Western blot analysis of wild type (+/+) or Atg7^-/-^ MEFs for the expression of Atg7, p62 and actin (loading control). (**B**) Measurement of oxygen consumption for WT and Atg7^-/- ^ MEFs under basal conditions, following the addition of the mitochondrial electron chain inhibitor oligomycin (0.5 μM), or in the presence of the mitochondrial uncoupler FCCP (1 μM), to determine maximal oxidative capacity.  Shown is the average fold change +/- SEM in oxygen consumption (WT MEFs basal respiration=1) obtained from 5 experiments each performed in triplicate. **(C**)  Assessment of mitochondrial number in WT or Atg7^-/-^ MEFs. DNA was isolated from WT (n=3 independent WT MEF cell isolates) and Atg7^-/-^ MEFs (n=3 independent Atg7^-/- ^MEF cell isolates) and quantitative PCR analysis performed for the mitochondrial-encoded gene ND1 and the nuclear-encoded gene H19.  (**D**) Relative extracellular acidification rates indicating lactic acid production and hence glyolytic rates in WT or Atg7^-/-^ MEFs.  Shown is the average +/- SEM fold change in lactic acid production from 8 experiments each performed in triplicate. * p≤0.05; ** p≤0.01.

In order to further characterize the defect in mitochondrial function within the context of intact cells, we next isolated mouse embryonic fibroblasts (MEFs) from wild type or Atg7^-/- ^embryos (Figure 2A). Compared to WT MEFs, Atg7^-/-^ MEFs exhibited a reduction in basal oxygen consumption (Figure 2B). In addition, we noted that Atg7^-/-^ MEFs demonstrated a marked reduction in maximal mitochondrial oxidative capacity, as assessed by the levels of FCCP-stimulated respiration. These differences were not a result of any apparent differences in overall mitochondrial numbers between the two cell types (Figure 2C). Coincident with this decrease in mitochondrial oxygen consumption, we noted that Atg7^-/- ^MEFs generated more lactic acid, consistent with an increase reliance on glycolysis (Figure 2D). This shift away from aerobic respiration and towards cytosolic glycolysis in Atg7^-/- ^MEFs presumably represents a compensatory mechanism to maintain intracellular energetic homeostasis in the setting of dysfunctional mitochondria.

**Figure 3.**
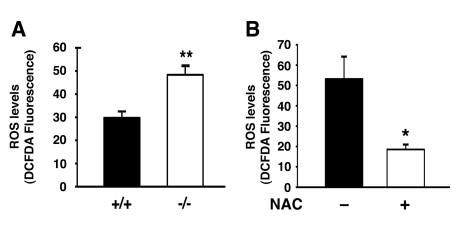
Atg7 deficient cells exhibit increased levels of ROS. (**A**) Intracellular ROS levels as assessed by DCFDA fluorescence intensity (arbitrary units) in WT and Atg7^-/- ^MEFs. ROS measurements were made from three independent WT or Atg7^-/- ^MEF primary cell isolates and the fluorescent intensity of more than 250 cells of each genotype were assessed.  **(B**) NAC treatment reduces the levels of ROS in MEFs lacking Atg7. Levels of ROS were assessed by DCFDA fluorescence in Atg7^-/-^ MEFs untreated or treated with NAC (500 μM) for 4 days prior to imaging. Values represent the normalized fluorescent intensity (arbitrary units) of approximately 300 cells per condition. Graphs represent the mean +/- SEM.

Damaged mitochondria often produce increased levels of reactive oxygen species (ROS). This increase in ROS can further increase mitochondrial damage leading in turn to more oxidant release and additional mitochondrial damage, in a process termed the ‘vicious cycle'[17]. In some circumstances, this perpetuating cycle of mitochondrial damage and oxidative stress is thought to contribute to normal aging as well as many age-related diseases [18]. Given the above observations, we next sought to assess whether continuous oxidative stress was evident in autophagy deficient cells. As noted in Figure 3A, Atg7^-/-^ MEFs had increased levels of intracellular ROS. Culturing these cells in the presence of the antioxidant N-acetylcysteine (NAC) resulted in a reduction in ROS levels (Figure 3B).

**Figure 4.**
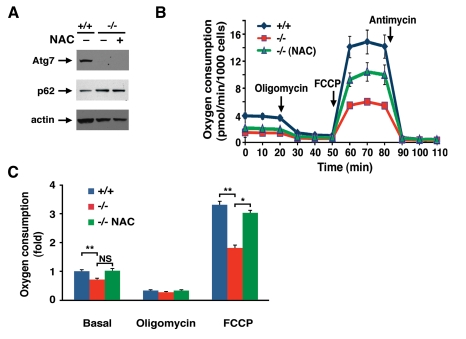
NAC treatment partially corrects the metabolic defect observed in Atg7 ^-/-^ MEFs. (**A)** Western blot analysis of wild type (+/+) or Atg7^-/-^ MEFs for the expression of Atg7, p62 and actin (loading control) cultured in the presence or absence on the antioxidant NAC (500 μM) for ten days. (**B)** Primary wild-type and Atg7^-/- ^MEFs that were cultured in the absence or presence of 500 μM NAC for 10 days prior to cellular respiration measurement.  Shown is a representative tracing of oxygen consumption performed in triplicate under basal conditions, following the addition of oligomycin (0.5 μM), the pharmacological uncoupler FCCP (1 μM) or the Complex III inhibitor antimycin A (0.25 μM). (**C)** Averaged metabolic profile from 4 separate experiments employing 3 independent primary isolates of WT and Atg7^-/- ^MEFs. Shown is the fold change +/- SEM in oxygen consumption (WT MEF basal respiration =1) for WT MEFs and for Atg7^-/-^ MEFs that were cultured in the absence or presence of 500 μM NAC for 10 days prior to metabolic assessment.* p≤0.05; ** p≤0.01; NS= not significant.

Antioxidant treatment did not appear to alter the level of autophagic flux in Atg7^-/-^ MEFs as the level of p62 was unaltered in NAC treated cell (Figure 4A). However, chronic NAC treatment did partially ameliorate the observed metabolic defect seen in these cells (Figure 4 B, C).  These results suggest that the continuous oxidative stress observed in Atg7^-/-^ MEFs contributes to the decline in mitochondrial function.

### Oxidative stress and glucose intolerance in pancreatic Atg7^-/-^ mice

**Figure 5.**
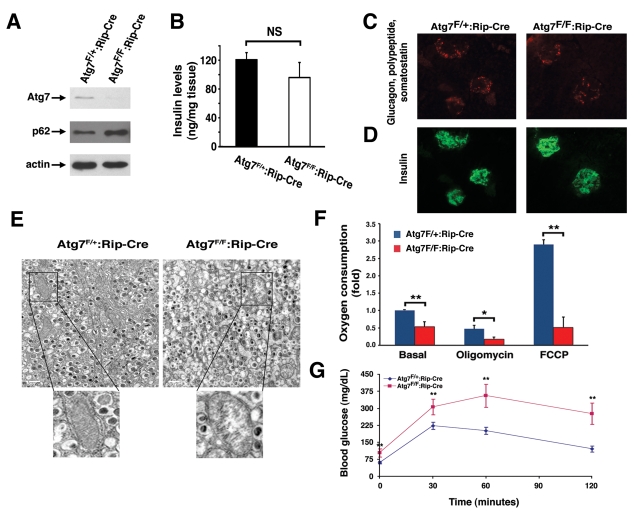
Mice deficient in Atg7 expression within pancreatic β cells demonstrate altered mitochondria. ** (A)** Western blot analysis of purified pancreatic islets obtained from Atg7^F/+^:Rip2-CRE or Atg7^F/F^:Rip2-CRE mice demonstrating the relative expression of Atg7, p62 and actin (loading control). (**B**) Intracellular insulin levels (mean +/- SEM) in pancreatic tissue of 8-9 week old Atg7^F/+^:Rip2-Cre (n=4 mice) or Atg7^F/F^:Rip2-Cre mice (n=5 mice). The slight reduction in insulin levels in the Atg7^F/F^:Rip2-Cre mice was not significant when compared to the control. (**C**) Pancreatic sections of control Atg7^F/+^:Rip2-Cre or Atg7^F/F^:Rip2-Cre mice were stained for non-β cell components within the islets with the simultaneous use of anti-glucagon, anti-somatostatin, and anti-polypeptide antibodies. (**D**) Serial sections were used to visualize β cells with an anti-insulin antibody.  Eight week old mice lacking autophagy in β cells have qualitatively similar levels of α, δ, and polypeptide producing cells within their islets, as well as similar levels of β cells when compared to control mice. (**E**) Electron micrographs demonstrating the accumulation of swollen, dysmorphic mitochondria within the Atg7-deficient β cells. (**F**) Isolated islets from control and Atg7^-/-^ mice were assessed for fold +/- SEM changes in basal respiration (Atg7^F/+^:Rip2-Cre isolated islets=1), and for oxygen consumption in the presence of oligomycin (0.5 μM) or FCCP (0.5 μM). Results are normalized to islet protein concentration and are from n=4 mice per genotype. (**G**) Impaired glucose tolerance in Atg7^F/F^:Rip2-Cre mice. Blood glucose measurements were made in 8-10 week-old control mice Atg7^F/+^:Rip2-Cre (n=10 mice) or Atg7^F/F^:Rip2-Cre mice  (n=8 mice) following the IP injection of D-glucose (1 g/kg).  Data represent the mean +/- SEM. *p≤0.05; **p≤0.01.

Given the profound alterations observed in isolated mitochondria derived from Atg7 deficient skeletal muscle and the observation that NAC treatment could at least partially reverse the metabolic defects observed in Atg7^-/-^ MEFs, we next sought to assess whether these principles could be applied to the physiological defects seen in an *in vivo *model of Atg7 deficiency. Since our skeletal muscle conditional Atg7^-/-^ mice did not exhibit an overt phenotype, we created an additional model in which Atg7 was deleted within pancreatic β cells by crossing the Atg7-floxed mice with RIP2-Cre animals. Western blot analysis from purified pancreatic islets demonstrated that conditional knockout animals (Atg7^F/F^:RIP2-Cre) had reduced or absent Atg7 expression and a corresponding increase in p62 levels (Figure 5A). In young mice, deletion of Atg7 within β cells did not result in significant alterations in pancreatic insulin expression (Figure 5B). Similarly, the cellular composition of  individual pancreatic islets was largely unperturbed in 8 week old mice (Figure 5 C, D). In contrast, electron micrographs of control or knockout tissues revealed the early accumulation of markedly abnormal mitochondria within the β cells of Atg7 deficient mice (Figure 5E). Analysis of basal and FCCP-stimulated respiration from isolated pancreatic islets revealed a significant decrease in basal mitochondrial respiration and a marked decrease inmitochondrial oxidative capacity in Atg7 deficient islets (Figure 5F). Consistent with previous reports describing animals with alterations in β cell mitochondria [12,13,19], mice lacking Atg7 within their β cells also exhibited marked abnormalities in glucose tolerance (Figure 5G).

**Figure 6.**
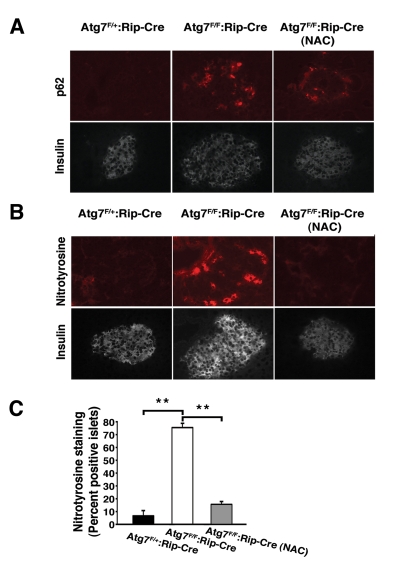
In vivo treatment with NAC reduces oxidative stress within pancreatic β cells. (**A**) Atg7^F/+^:Rip2-Cre or Atg7^F/F^:Rip2-Cre mice that were untreated or treated with the antioxidant NAC for 12 weeks.  At 16 weeks of age, mice were sacrificed and serial sections of pancreatic tissue were analyzed for p62 and insulin or (**B**) nitrotyrosine and insulin. (**C**) Quantification of nitrotyrosine staining in islets of control mice, Atg7-deficient animals or Atg7-deficient mice treated for 12 weeks with NAC (n=3 animals per group). Graph represents the mean+/- SEM. **; *p*≤0.01.

We next asked what the role of continuous oxidative stress was in this model of β cell dysfunction. We randomized knockout or control mice beginning at age 4 weeks to treatment with or without NAC.  As expected, when compared to control animals, mice with conditional ablation of Atg7 accumulated increased levels of p62 within their islets (Figure 6A). Treatment with NAC did not noticeably affect this accumulation in conditionally ablated animals (0/18 islets p62 positive in Atg7^F/+^:RIP2-Cre mice; 22/24 islets p62 positive in Atg7^F/F^:RIP2-Cre mice and 17/17 islets p62 positive in Atg7^F/F^: RIP2-Cre mice treated with NAC; in random slides obtained from n=3 mice per condition). In addition, as an *in situ* marker of oxidative stress, we measured nitrotyrosine levels which are known to increase and contribute to the diabetic phenotype [20]. As noted, levels of nitrotyrosine were markedly elevated in Atg7 deficient islets and in contrast to our observations with p62, treatment with NAC was very effective in reducing the observed increase (Figure 6 B, C).

**Figure 7.**
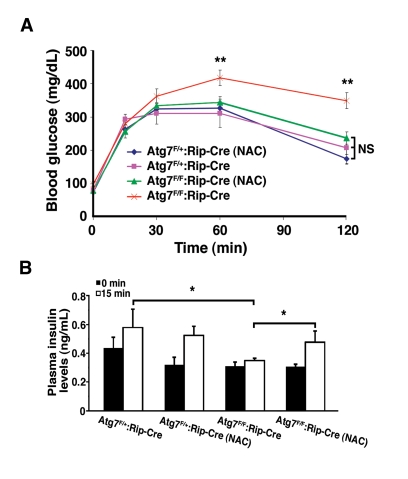
NAC treatment of Atg7 deficient mice prevents the development of a glucose intolerance phenotype. (**A)** Male Atg7^F/+^:Rip2-Cre (n=6 mice), Atg7^F/+^:Rip2-Cre (+NAC; n=6 mice), Atg7^F/F^:Rip2-Cre (n=11 mice) or Atg7^F/F^:Rip2-Cre (+NAC; n=11 mice) were fasted overnight and subsequently injected with 1 g/kg D-glucose. Serum glucose levels were measured and the untreated β cell Atg7 deficient mice were found to be statistically different at the indicated time points, while the other three groups of mice were statistically indistinguishable over the 2 hr timecourse. (**B)** Insulin levels were determined by tail vein blood sampling at time 0 and 15 min following glucose administration: Atg7^F/+^:Rip2-Cre (n=6 mice), Atg7^F/+^:Rip2-Cre (+NAC; n=4 mice), Atg7^F/F^:Rip2-Cre (n=8 mice) or Atg7^F/F^:Rip2-Cre (+NAC; n=5 mice).  Data are represented as the mean +/- SEM.  * p≤0.05; ** p≤0.001.

We next asked whether reducing the levels of oxidative stress by itself was sufficient to ameliorate the physiological impairment observed with conditional deletion of Atg7.  Littermates were randomized after weaning to treatment with or without NAC and subsequently assessed at age 16 weeks. Consistent with continuous oxidative stress playing a causative role in the underlying physiology, and in contrast to untreated Atg7^F/F^:RIP2-Cre mice,  NAC treated Atg7^F/F^:RIP2-Cre mice had a glucose tolerance response that was indistinguishable from control mice (Figure 7A). This protection was also seen at later time points, although the overall degree of rescue appeared to be reduced as the mice aged (data not shown). The observed differences in glucose homeostasis were not a result of reduced peripheral insulin sensitivity as insulin tolerance tests were comparable for all four groups tested (data not shown).  Furthermore, mice lacking Atg7 within their β cells develop a defect in glucose-stimulated insulin secretion, and this defect was not observed in conditionally ablated mice treated with an antioxidant (Figure 7B).

## Discussion

Using a variety of cellular and *in vivo* models, we demonstrate that impairment of autophagy leads to the accumulation of damaged and dysfunctional mitochondria and a corresponding increase in intracellular ROS levels. In a model of autophagy deficiency occurring within the pancreatic β cell, we further  demonstrate  that the overall  physiological impairment in glucose tolerance and insulin secretion can be significantly ameliorated by the simple addition of an antioxidant. While assessment of autophagic flux by p62 expression suggests that NAC treatment does not directly affect autophagy, the ultimate improvement of glucose tolerance and glucose-stimulated insulin secretion suggests that at least for this *in vivo* model, continuous oxidative stress plays an important pathophysiological role.

A very recent report has suggested that oxidative stress may also play a role in the alterations in innate immunity observed in autophagy deficient cells [21]. In particular, ROS appeared to mediate the increase in interferon secretion and resistance to viral infection seen in Atg5^-/-^ cells. The form of autophagy studied in these experiments is quite specialized and involves the delivery of viral nucleic acids to the endosome rather than the standard situation where cargo is delivered to the lysosome. Similarly, it should be noted that in this context, Atg5 deficient cells demonstrate increased interferon protection and increased protection from viral infection rather than the usual situation where autophagy disruption results in a loss of function phenotype. Interestingly, in this recent study, Atg5^-/-^ MEFs exhibited an approximate 2-fold increase in the number of  mitochondrial genomes per cell, while for presently unclear reasons, we did not observe a similar increase in mitochondrial number in our Atg7^-/-^ MEFs (see Figure 2C).

Based on these recent *in vitro* observations regarding Atg5 deletion in cells and our *in vivo* observations regarding Atg7 deletion in the pancreas, it is tempting to speculate that a rise in ROS levels may be a universal downstream mediator of the positive or negative alterations seen in autophagy deficient tissues. Nonetheless, other evidence suggests that such a broad conclusion is unlikely to be always correct. For instance, while the accumulation of ubiquitin positive protein aggregates in inclusion bodies are prominent features in Atg5 and Atg7 deficient neurons and cardiomyocytes, these changes are not observed in T lymphocytes or dendritic cells that lack Atg5 expression [10, 11, 14, 22, 23]. This may reflect fundamental differences in the role of autophagy in rapidly dividing versus postmitotic cells.  Similarly, even within a single tissue or organ, the effects of abrogating or inhibiting autophagy cannot always be easily predicted. For instance, deletion of Atg5 in adult mice leads to the spontaneous appearance of contractile dysfunction, while the same deletion performed early in cardiogenesis does not result in any basal myocardial phenotype [14]. Even less straightforward are observations that while cardiac specific deletion of Atg5 results in an animal that is less able to withstand myocardial pressure overload, heterozygous deletion of beclin, another essential autophagy gene, results in mice with the seemingly opposite cardiac phenotype [14, 24]. Thus, the downstream mediators and ultimate consequences of inhibiting autophagy may vary widely depending on the strategy used to disrupt autophagic flux and in what tissue or organ the disruption occurs.

Our data suggests that antioxidant treatment with NAC is particularly beneficial in preventing the glucose intolerance phenotype observed following deletion of Atg7 within β cells. Pancreatic secretion of insulin is well known to be sensitive to changes in the cellular redox state and overall mitochondrial function. Given our results on the metabolic profile of Atg7^-/- ^MEFs cultured in the presence of NAC, it is tempting to think that in the setting of Atg7 deletion, the *in vivo* use of antioxidants interrupts the ‘vicious cycle' of mitochondrial generated ROS inducing further mitochondrial damage. These observations suggest that antioxidant targeted therapy might be beneficial for at least a subset of the growing number of conditions where deficiency or impairment of autophagy is thought to contribute.

## Methods

Mice and cells. Atg7^F/F^ mice have been previously described [9] and were crossed with either MCK-Cre mice (Jackson Laboratory) or RIP2-Cre mice (Jackson Laboratory) to generate mice with a conditional deletion of Atg7 within skeletal muscle or pancreatic β cells. For genotype analysis, tissues were digested overnightat 55 °C with Gitschier buffer (67 mM Tris-HCL,pH 8.8, 16.6 mM ammonium sulfate, 6.5 mM MgCl_2_, 0.5% TritonX-100, 1% - mercaptoethanol, 100 μg/ml proteinase K). Samples were incubated at 95 °C for 5 min, shaken for 20 min with an Eppendorf thermomixer, and centrifuged at 16,100 x *g* for 2 min. Three primers 5'- TGGCTGCTACTTCTGCAA TGA TGT-3', 5'- GCAAGCTCACTAGGC TG CAGAACC-3', and 5'-GGTCCA GAGTCCGGTCTC GG-3' were used to detect the flox Atg7 allele in genomic DNA.

Cre-mediated recombination was assessed by PCR analysis of genomic DNA using primers 5'- GGTCTGGCAG TAAAAACTATC-3' and 5'-GTGA AACAGCATTGCTGTCACTT-3', and 5'-GGGTCC CA AAGGCCGCC-3' and 5'-GGATAGTTTTTACT GCCAGAC CGC-3' for Rip2-CRE and  MCK-CRE, respectively [25, 26]. Similar to what has been recently described [12, 13], we observed no obvious phenotypic differences between Atg7^+/+^, Atg7^+/+^: MCK- Cre, Atg7^+/+^:Rip2-Cre, Atg7^F/F^, Atg7^F/+^:MCK-Cre or Atg7^F/+^:RIP-Cre mice, and so we predominantly present the latter two genotypes as representative controls in this study.

For chronic NAC treatment, randomized 4- week old mice were treated with 1 g/L of NAC in the drinking water for the indicated duration of the study. All animal experiments were conducted in accordance with the guidelines of the Animal Care and Use Committee, National Heart Lung and Blood Institute, NIH.

Mouse embryonic fibroblasts were prepared from E12-E14 day old embryos using standard methods. MEFs were prepared following a cross between Atg7^F/F ^mice with a transgenic mouse line carrying the Cre recombinase under the control of the adenoviral promoter EIIa that is known to be broadly expressed in the developing embryo [27].  PCR analyses using primers, 5'- GCTG CTACTTC TGCAATGATGT-3' and 5'GCAAGCTCACTAGG CTGCAGAACC-3', were used to detect the wild-type Atg7 gene and primers, 5'- GCTGCTAC  TTCTGCAAT GATGT-3' and 5'- ATG GTAC ATGCTAAGCCTCTGGAC-3', were used to detect the deleted Atg7 gene.  MEFs were cultured in growth medium consisting of Dulbecco's Modified Eagle's medium (DMEM; Invitrogen) supplementedwith 15% fetal bovine serum (FBS), 50 units/ml penicillin and 50 μg/ml streptomycin.  For *in vitro* NAC treatment, freshly thawed primary MEFs at passage 2 were placed in growth medium supplemented with 500 μM NAC for 10 days with media being changed every other day.

Glucose tolerance test, insulin measurement and islet isolation. Overnight fasted male mice were given intraperitoneal injections of D-glucose (20% solution; 1 g/kg body weight) and blood glucose was determined using a one-touch Ascensia Elite glucometer (Fisher). Tail vein blood was collected at 0 and 15 min following glucose injection and plasma insulin levels were measured with a rat insulin radioimmunoassay kit according to the manufacturer's recommendations (Millipore).

For pancreatic intracellular insulin measurement, small sections of pancreas were digested with ethanol/acid buffer (25 ml absolute ethanol, 8.3 ml ddH_2_O, and 0.5 ml concentrated HCl) overnight at 4°C as previously described [30].  Insulin was measured with a Rat/Mouse Insulin ELISA Kit Insulin (Crystal Chem Inc.). Tissue weight was used to normalize the intracellular insulin levels.

Pancreatic islets were isolated using a standard protocol [31].  Briefly, the pancreas was perfused with a solution of 0.5 mg/ml Collagenase Type V (Sigma) dissolved in Hanks Balanced Salt Solution (HBSS) containing calcium and magnesium (Mediatech Inc).  The digestion was performed at 37°C for 15-20 min after which the collagenase was neutralized with HBSS supplemented with 1% FBS. The collagenase treated pancreas was then sequentially filtered through 1.5 mm and 0.8 mm metal mesh filters.  Islets were subsequently enriched by centrifugation in Histopaque 1077 (Sigma) and hand-picked under direct light microscopic visualization.

Isolated mitochondrial and intact metabolic studies . Purified mitochondria were isolated from freshly harvested skeletal muscle using standard methods [28]. Briefly, isolated skeletal muscle were rapidly harvested, washed and minced in ice-cold Ionic Medium (100 mM surcrose, 10 mM EDTA, 100 mM Tris-HCL, 46 mM KCl, pH 7.4). The tissues were digested with 5% Proteinase Type XXIV (Sigma) in Ionic Medium for 5 min on ice and the protease was subsequently inactivated by the addition of Ionic Medium supplemented with 0.5% BSA.  Samples were then homogenizedwith a glass-Teflon motorized homogenizer and the mitochondrial fraction isolated by differential centrifugation. Mitochondria were subsequently washed twice and then resuspended in Suspension Medium (230 mM mannitol, 70 mM sucrose, 0.02 mM EDTA, 20 mM Tris-HCl, 5 mM K_2_HPO_4_, pH 7.4) prior to functional assessment.   Succinate (5 mM), rotenone (1 μM), Antimycin A (0.25 μM) and ADP (1 mM) were used to assay complex II dependent respiration.

Measurement of intact cellular respiration was performed using the Seahorse XF24 analyzer (Seahorse Bioscience Inc.). Primary MEFs were plated at a density of 40,000 cells/well on XF24 tissue culture plate.  Purified pancreatic islets were isolated and cultured overnight in DMEM supplemented with 10% FBS, 50 units/ml penicillin and 50 μg/ml streptomycin. Two-three hours prior to respiration measurements, 50-100 islets were transferred to poly-L-lysine coated XF24 tissue culture plate.  Prior to the respiration assay, primary MEFs or islets were rinsed and cultured in DMEM running medium (8.3g/L DMEM (Sigma), 200 mM GlutaMax-1 (Invitrogen), 100mM sodium pyruvate (Sigma),  25 mM D-glucose (Sigma), 63.3 mM NaCl (Sigma), and phenol red (Sigma), adjust pH to 7.4 with NaOH) according to manufacturer's protocol.  Oxygen consumption was measured under basal conditions, in the presence of the mitochondrial inhibitors oligomycin (0.5 μM) or antimycin A (0.25 μM), or in the presence of the mitochondrial uncoupler FCCP (0.5 μM or 1 μM) to assess maximal oxidative capacity. Lactate measurements were made by determining the change in extracellular pH over time, as previously described [29].  Oxygen consumption and lactate measurements were normalized to cell number for primary MEFs and protein concentration for pancreatic islets.

Histology and Immunohistochemistry. Histological analysis was performed on 8-10 week old and 16-24 week old mice. Skeletal muscle or pancreatic tissue was isolated from Atg7^+/+^:MCK-Cre, Atg7^F/+^:MCK-Cre, and Atg7^F/F^:MCK-Cre, or Atg7^+/+^:RIP2-Cre, Atg7^F/+^:RIP2-Cre, and Atg7^F/F^:RIP2-Cre mice, respectively.   Tissues were fixed in 10% formalin and paraffin embedded tissue sections were used for subsequent hematoxylin and eosin (H&E) staining. Immunohistochemistry analysis with anti-insulin (DAKO), anti-glucagon (Sigma), anti-somatostatin (DAKO), and antipolypeptide (Millipore) antibodies was performed using standard protocols. Cryostat sections were fixed with 4% paraformaldehyde in PBS prior to immunohistochemical analysis with either a nitrotyrosine antibody (Millipore) or p62 antibody (Progen Biotechnik).  For quantification purposes, an islet was considered to stain positive for p62 or nitrotyrosine if greater than 5 cells within the islet exhibited positive staining. Rhodamine or Cy2 conjugated secondary antibodies (Jackson Immunological Research Laboratories) were used for visualization.  Electron micrographs were performed on ultrathin sections of tissues that were fixed in 2 or 2.5% glutaraldehye plus 1% paraformaldehyde, post-fixed with 1% OsO_4_, stained en bloc with 1% uranyl acetate and embedded in Embed 812 (Electron Microscopy Sciences) using standard methods. The sections were stained with lead citrate and uranyl acetate before viewing.

Western Blot Analysis. Primary MEFs or isolated pancreatic islets were lysed with NonidetP-40 lysis buffer (1.0% Nonidet P-40, 50 mM Tris-HCl pH 7.4,150 mM NaCl, 5 mM EDTA) supplemented with protease inhibitor tablet (Roche) and phosphatase inhibitors (1 mM Na_3_VO_3_, 1 mM β-glycerolphosphate, 10 mM NaF) for 15 min on ice prior to clarificationby centrifugation at 16,100 x *g* for 15 min at 4 °C.  Skeletal muscle was suspended in homogenizing buffer (25 mM Tris. HCl pH 7.4, 150 mM NaCl, 5 mM EDTA, 1% Triton X-100, 0.1% SDS, 10% glycerol, 1 mM DTT, 0.5 % sodium deoxycholic acid) supplemented with protease and phosphatase inhibitors and homogenized using a tissue lyser (Qiagen). Protein concentration was determined with the Pierce BCA assay Kit (Thermo Fisher Scientific).  Protein lysates were resolved on precast Tris-Glycine SDS gels (Invitrogen) and transferred onto nitrocellulose. Immunoblot analysis was performed with antibodies directed against Atg7 (ProSci Inc and Sigma), actin (Sigma), the Complex I component NDFS3 (NADH-ubiquinone oxidoreductase, MitoSciences), the Complex II component FP (flavoprotein subunit of complex II, MitoSciences), and the Complex III component Core1 (Ubiquinol-cytochrome-c reductase complex core protein 1, MitoSciences).

For detection of mitochondria complexes on native blue gels, purified mitochondria from skeletal muscle were resolved on Native PAGE Novex Bis-Tris gels according to the manufacturer's instructions (Invitrogen).  Briefly, 1 mg of a purified mitochondria pellet was resuspended with 100 μl of 1x NativePAGE buffer and 12.5 μl of 10% maltoside (Sigma).  The samples were incubated on ice for 30 min with frequent vortexing before pelleting by centrifugation at 16,100 x *g* for 10 min at 4°C.  After centrifugation, 100 μl of supernatant was transferred to a new tube containing 6.3 μl of Coomassie blue additive and samples (approximately 50 μg) were then resolved by Native PAGE Novex Bis-Tris gel electrophoresis.

ROS measurements. Primary MEFs were cultured on Nunc Lab-Tek two chamber glass slides.  As an internal control, each dual chamber slide contained one well of WT MEFs and one well of Atg7^-/- ^MEFs, or one well of Atg7^-/-^ MEFs and one well of Atg7^-/- ^ MEFs that have been treated with 500 μM NAC for 4 days.  Cells were incubated with HBSS containing 50 μM 5-(and-6)-chloromethyl-2',7'-dichlorodihydrofluorescein diacetate, acetyl ester (CM-H_2_DCFDA; Invitrogen) for 30 min at 37°C.  Cells were then rinsed and mounted with mounting medium (Vector Labs) and visualized with a Leica SP1 confocal microscope as previously described [32].  Several random fields were taken of each genotype and mean fluorescence intensity was calculated in three or more separate experiments with data from over 250 cells using at least two independently isolated WT and Atg7^-/-^ MEF cell isolates. Attached cells were measured rather than MEF cell suspensions since we have previously observed dramatic alterations in ROS levels when attached cells are trypsinized [33].

Mitochondrial number. The ratio of mitochondrial DNA to nuclear DNA was determined as previously described [34]. In brief, quantitative PCR analysis using SYBR green (Applied Biosystems) was performed with 25 μg of isolated DNA (Qiagen).   Mitochondrial DNA was assessed using primers, 5'-CTCTTAT CCACGC TTCCGTTACG-3' and 5'-GATGGTGGTACTCCCGCT GTA-3' for the mitochondrial-encoded ND1 gene.  Nuclear DNA level was determined by amplifying the genomic H19 locus using primers 5'-GTACCCACCTGTCGTCC-3'and 5'-GTCCACGAGACCAATGA CTG-3'.  The relative amount of mitochondrial to nuclear DNA was determined by normalized ND1 to H19 levels.
